# Ubiquitin Ligase NEDD4 Regulates PPARγ Stability and Adipocyte Differentiation in 3T3-L1 Cells

**DOI:** 10.1038/srep38550

**Published:** 2016-12-05

**Authors:** Jing Jing Li, Ruishan Wang, Rati Lama, Xinjiang Wang, Z. Elizabeth Floyd, Edwards A. Park, Francesca-Fang Liao

**Affiliations:** 1 Department of Pharmacology, University of Tennessee Health Science Center, Memphis, Tennessee 38103, USA; 2 Department of Pharmacology & Therapeutics, Roswell Park Cancer Institute, Buffalo, NY 14263, USA; 3 Ubiquitin Biology Laboratory, Pennington Biomedical Research Center, Louisiana State University Systems, Baton Rouge, LA 70808, USA; 4 Department of Veterans Affairs Medical Center, Memphis, Tennessee 38104, USA

## Abstract

Peroxisome proliferator–activated receptor-γ (PPARγ) is a ligand-activated nuclear receptor which controls lipid and glucose metabolism. It is also the master regulator of adipogenesis. In adipocytes, ligand-dependent PPARγ activation is associated with proteasomal degradation; therefore, regulation of PPARγ degradation may modulate its transcriptional activity. Here, we show that neural precursor cell expressed developmentally down-regulated protein 4 (NEDD4), an E3 ubiquitin ligase, interacts with the hinge and ligand binding domains of PPARγ and is a bona fide E3 ligase for PPARγ. NEDD4 increases PPARγ stability through the inhibition of its proteasomal degradation. Knockdown of NEDD4 in 3T3-L1 adipocytes reduces PPARγ protein levels and suppresses adipocyte conversion. PPARγ correlates positively with NEDD4 in obese adipose tissue. Together, these findings support NEDD4 as a novel regulator of adipogenesis by modulating the stability of PPARγ.

Peroxisome proliferator–activated receptor γ (PPARγ) is a nuclear hormone receptor which is activated by its endogenous ligands, such as fatty acids and eicosanoids^1^. Pharmacologically, activation of PPARγ by the thiazolidinedione (TZD) class of insulin-sensitizing agents is associated with side effects including weight gain^2^. PPARγ activation regulates gene networks that are critically involved in inflammation^3^, adipocyte differentiation^4^, lipid metabolism^5^ and glucose homeostasis^6^.

The complex process of adipocyte differentiation from preadipocytes is orchestrated by PPARγ and the CCAAT/enhancer-binding protein (C/EBP) family transcription factors. PPARγ in particular has been considered to be the master regulator of adipogenesis^7^. The two isoforms of PPARγ, the more widely expressed γ1 and the adipocyte-specific γ2, differ only in 30 amino acids at the N termini^8^. Like most nuclear receptors, PPARγ contains a ligand-independent transactivation domain termed Activation Function 1 (AF-1), a conserved central region DNA-binding domain (DBD), a hinge region, and a C-terminal ligand-binding domain (LBD). PPARγ2 is generally believed to play a more central role in adipogenesis though both isoforms are important during this process^9^,^10^,^11^.

In adipocytes, PPARγ activation is linked to proteasomal degradation^12^,^13^,^14^. Therefore, regulation of PPARγ degradation may provide novel regulatory mechanisms of its transcriptional activity. Recently, several PPARγ ubiquitin-protein ligases (E3s) have been identified in adipocytes^15^,^16^,^17^. While seven in absentia homolog 2 (SIAH2)^16^ and makorin ring finger protein 1 (MKRN1)^15^ cause PPARγ degradation, triparite motif protein 23 (TRIM23) regulates PPARγ ubiquitination to stabilize it ref. 17. These observations demonstrate important roles for E3 ligases in PPARγ posttranslational regulation.

Neural precursor cell expressed developmentally down-regulated protein 4 (NEDD4), a Homologous to the E6-AP Carboxyl Terminus (HECT)-type E3 ubiquitin ligase, is the prototypical member in NEDD4 family of proteins. These proteins have conserved roles in mediating ubiquitin-dependent trafficking and/or degradation of plasma membrane proteins^18^. Our previous studies have shown that mice heterozygous for NEDD4 were less obese after feeding them a high-fat diet (HFD)^19^. Here, we identified NEDD4 as a novel PPARγ interacting protein. Our data suggest that NEDD4 directly ubiquitinates PPARγ and increases its stability through the inhibition of its proteasomal degradation.

## Results

### NEDD4 interacts with PPARγ

**Figure 1:**
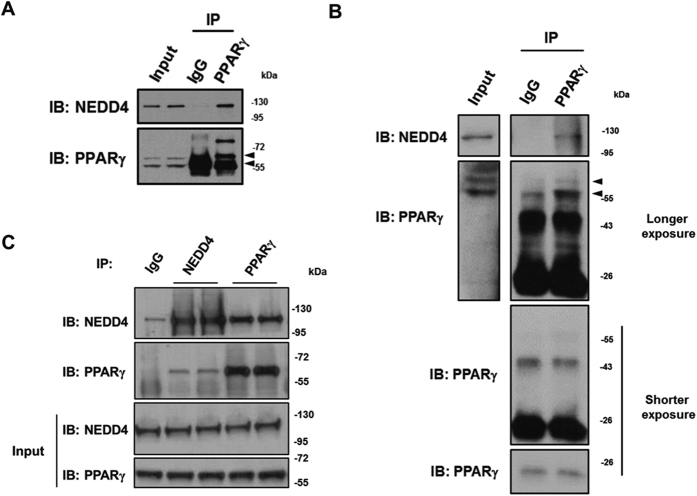
NEDD4 interacts with PPARγ. (**A**) Interaction between NEDD4 and PPARγ in 3T3-L1 adipocytes. Total lysates (500 μg) were immunoprecipitated with 1 μg of anti-PPARγ antibody (clone E-8). Normal mouse IgG served as negative control. (**B**) *In vivo* interaction between NEDD4 and PPARγ. Epididymal adipose tissue lysates (500 μg) were immunoprecipitated with 1 μg of anti-PPARγ antibody (clone E-8) and immunoblotted with anti-NEDD4 antibody. PPARγ immunoblot was performed with clone T.647.5. (**C**) Co-immunoprecipitation of NEDD4 and PPARγ in HEK293 cells transiently expressing plasmids containing T7-tagged NEDD4 and FLAG-tagged PPARγ2. The rabbit monoclonal anti-PPARγ antibody (clone T.647.5) was used for immunoprecipitation, and the mouse monoclonal anti-PPARγ antibody (clone E-8) was used for immunoblotting. Lysates pulled down with normal rabbit IgG served as negative control. Full-length blots are presented in Supplementary Fig. S4.

**Figure 2:**
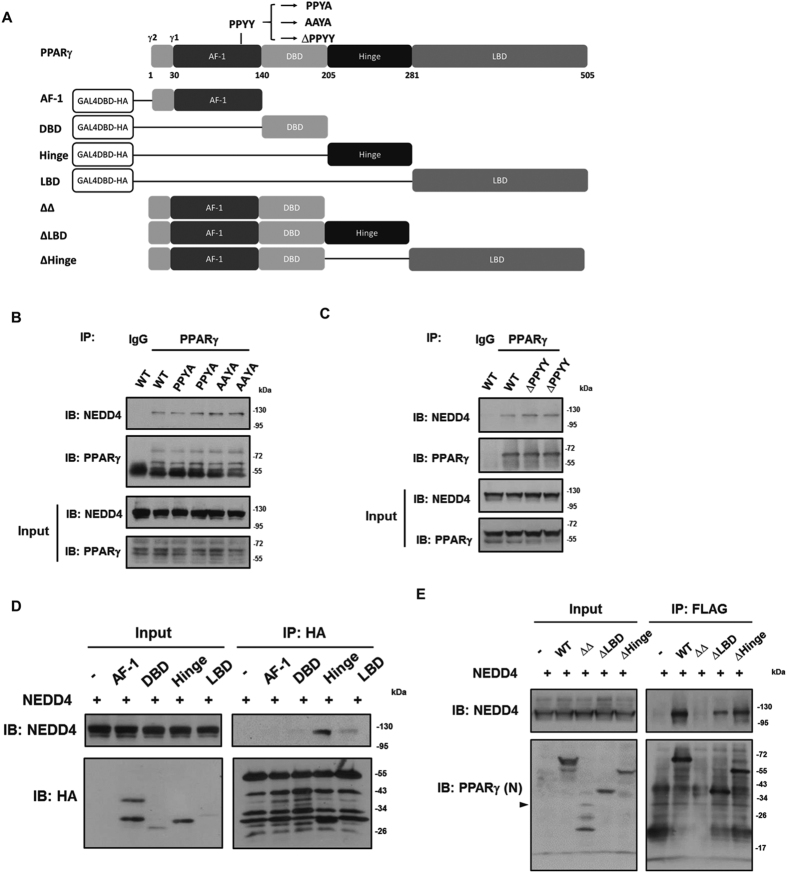
NEDD4 associates with the hinge/ligand binding domain of PPARγ. (**A**) Schematic drawings of PPARγ protein domains and domain-deleted mutants. GAL4DBD-HA plasmid was fused with AF-1, DBD, Hinge, or LBD domain of mouse PPARγ2. (**B**,**C**) The PPxY motif is not required for NEDD4-PPARγ interaction. HEK293 cells were transiently co-transfected with T7‐tagged NEDD4 and FLAG‐tagged PPARγ2 or its mutants. The mouse anti-PPARγ antibody was used for immunoprecipitation. For immunoblotting, the mouse anti-PPARγ (clone E-8) antibody was used in panel B, while the rabbit anti-PPARγ (clone T.647.5) antibody was used in panel C. The IgG heavy chain was thus not detected in panel C. (**D**) NEDD4 interacts with the hinge and LBD domains of PPARγ. GAL4DBD-HA plasmids containing AF-1, DBD, Hinge, or LBD domain of PPARγ2 were co-expressed in HEK293 cells with T7‐NEDD4. Lysates were immunoprecipitated with anti-HA antibody. (**E**) Interaction between NEDD4 and domain-deleted FLAG-PPARγ2. HEK293 cells were transfected with T7-NEDD4 alone, or together with FLAG-PPARγ2, FLAG-PPARγ2∆Hinge (without amino acids 206–280), FLAG-PPARγ2∆LBD (without amino acids 281–505), or FLAG-PPARγ2∆∆ (without amino acids 206–505). Cells were harvested 48 hours after transfection. MG132 (10 μM) was added to the cells 16 hr before harvesting. Lysates were immunoprecipitated with anti-FLAG antibody. The rabbit anti-PPARγ (clone H-100) antibody against N-terminal PPARγ was used for immunoblotting. Arrowhead indicates expected size of the ∆∆ mutant. Full-length blots are presented in Supplementary Fig. S4.

We identified a highly conserved Proline-Proline-x-Tyrosine (PPxY) motif within PPARγ which could serve as a binding site for the WW domains of NEDD4. Using co-immunoprecipitation (co-IP) approaches, we found that the endogenous NEDD4 and PPARγ proteins could be pulled down together in 3T3-L1 cells, mouse fat tissue lysates, and HEK293 cells transiently overexpressing NEDD4 and PPARγ2 cDNAs (Fig. 1A–C). To determine if NEDD4 associates with PPARγ through the PPxY motif, we generated PPARγ2 mutants with the PPYY sequence being mutated to PPYA or AAYA, and a PPYY-sequence-deleted mutant (Fig. 2A). Surprisingly, interactions were still detected when PPARγ2 mutants were expressed (Fig. 2B,C), suggesting that a non-canonical binding exists between NEDD4 and PPARγ which is independent of the PPxY motif. To further delineate the binding site, we expressed cDNAs containing the four PPARγ2 domains (AF-1, DBD, Hinge, and LBD) in HEK293 cells. Co-IP results showed that NEDD4 binds the hinge and LBD domains of PPARγ (Fig. 2D). The interaction was less apparent with the LBD than with the hinge domain, possibly due to the lower expression of the GAL4DBD-HA-LBD plasmid (Fig. 2D input lane 5 vs. lane 4). Deletion of LBD (∆LBD) or hinge (∆Hinge) attenuated the interaction between NEDD4 and PPARγ2 (Fig. 2E). Interestingly, the mutant with both LBD and hinge domains (∆∆) being deleted displayed multiple unexpected bands and their sizes were much smaller than expected (Fig. 2E input lane 3, arrow indicates the expected size of ∆∆), suggesting that ∆∆ mutant is degraded when expressed in HEK293 cells. Possibly due to protein degradation of the ∆∆ mutant, we failed to pull down the protein with the use of an anti-FLAG antibody (Fig. 2E IP lane 3).

### NEDD4 is an E3 ligase for PPARγ

**Figure 3:**
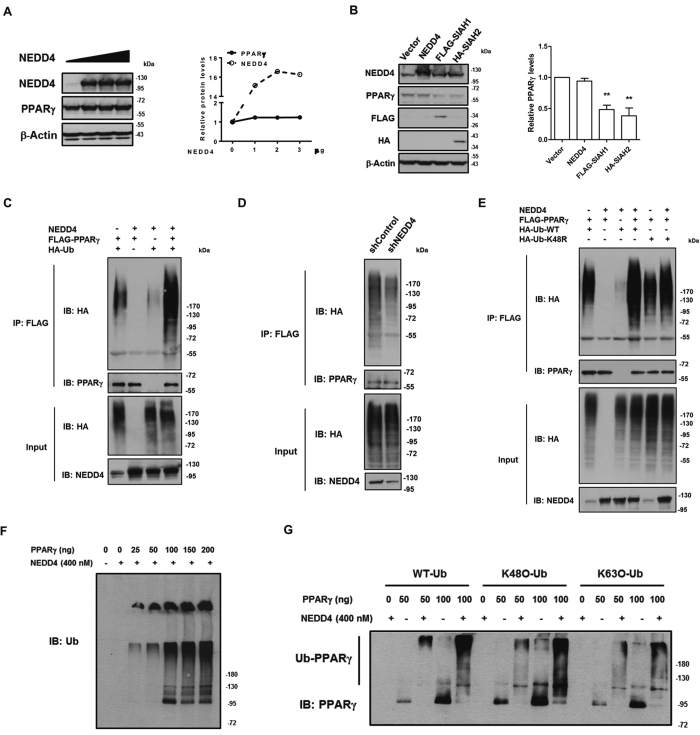
NEDD4 mediates lysine-48 independent ubiquitination of PPARγ. (**A**) Dose-dependent expression of NEDD4 does not reduce steady-state PPARγ protein abundance. Quantification of NEDD4 and PPARγ levels is on the right. Variable amounts of pRc-CMV-T7-NEDD4 expression vector from 1 to 3 μg were transfected with the PPARγ into HEK293 cells. NEDD4 plasmid expression saturates at 2 μg per well of a 6-well plate. (**B**) 1 μg of either FLAG-tagged SIAH1 or HA-tagged SIAH2, together with 1 μg of T7-tagged NEDD4, were transfected into CHO cells stably expressing FLAG-tagged PPARγ2. The relative PPARγ levels are quantified on the right. (**C**) NEDD4 overexpression enhances *in vivo* ubiquitination of PPARγ. T7-tagged NEDD4, FLAG-tagged PPARγ2, and HA-tagged ubiquitin (Ub) were transiently expressed in HEK293 cells at a ratio of 1.5:1:2 in combinations as indicated above the blots. (**D**) NEDD4 knockdown reduces *in vivo* ubiquitination of PPARγ. HEK293 cells seeded on 6-well plates were transfected with 1 μg of FLAG-tagged PPARγ2, 2 μg of HA-tagged Ub, along with 1.5 μg of plasmid containing NEDD4-targeting shRNA or non-targeting control shRNA per well. Cells were harvested for Western analysis 48 hr after the transfection. (**E**) NEDD4 overexpression enhances lysine-48 independent ubiquitination of PPARγ. T7-tagged NEDD4, FLAG-tagged PPARγ2, along with HA-tagged wild-type (WT) Ub or HA-tagged K48R Ub were transfected into HEK293 cells at a ratio of 1.5:1:2 in combinations as indicated above the blots. Western assays were performed at 48 hr after the transfection. (**F**) *In vitro* ubiquitination assay between NEDD4 and escalating amounts of recombinant His-GST tagged PPARγ2. (**G**) *In vitro* PPARγ2 ubiquitination by NEDD4 in the presence of wild-type ubiquitin (WT-Ub), K48-only ubiquitin (K48O-Ub), or K63-only (K63O-Ub). Data represent mean ± SEM of 3 independent experiments; *P < 0.05, **P < 0.01. Full-length blots are presented in Supplementary Fig. S4.

NEDD4 is an E3 ubiquitin ligase which typically facilitates proteasomal degradation of its substrates. Strikingly, we found that overexpression of NEDD4 in a dose-dependent manner did not alter steady-state PPARγ2 levels (Fig. 3A). Overexpression of SIAH1 or SIAH2—two highly homologous RING finger ubiquitin ligases—but not NEDD4, reduced PPARγ2 protein levels (Fig. 3B).

It has been demonstrated in several studies that PPARγ is subjected to ubiquitination^12^,^20^,^21^. To examine whether the ubiquitination of PPARγ was affected by NEDD4, we co-transfected HEK293 cells with different combinations of FLAG-tagged PPARγ2, T7-tagged NEDD4 and HA-tagged wild-type ubiquitin. PPARγ2 ubiquitination was detected in the presence of HA-tagged wild-type ubiquitin, and was significantly enhanced when exogenous NEDD4 was introduced (Fig. 3C). In contrast, shRNA-mediated knockdown of NEDD4 decreased PPARγ2 ubiquitination (Fig. 3D), suggesting that NEDD4 functions as an E3 ligase for PPARγ to promote its ubiquitin chain formation. Because NEDD4 does not reduce steady-state PPARγ expression, we reasoned that NEDD4 may facilitate proteasomal-independent ubiquitination of PPARγ. Among the seven lysines present in a ubiquitin molecule, K48-linked ubiquitin chains are generally known to label proteins for proteasomal degradation, while ubiquitin chains linked through other lysine residues (K6, K11, K27, K29, K33 and K63) or mixed chains may be involved in various cellular signaling and pathways not related to protein degradation^22^. To test if NEDD4 promotes the assembly of non-K48 linkages to PPARγ, we employed an HA-tagged K48R mutant ubiquitin with a single lysine to arginine mutation at position 48, to prevent the assembly of K48-ubiquitin chain. The anti-HA immunoblotting of PPARγ2 immunoprecipitated from HEK293 cell lysates containing co-expressed FLAG-tagged PPARγ2 and T7-tagged NEDD4 in the presence of either HA-tagged wild-type ubiquitin or K48R mutant revealed robust ubiquitinated forms of PPARγ2 at comparable levels (Fig. 3E). The basal ubiquitinated forms of PPARγ2 were slightly less in the presence of the K48R mutant compared to that in the presence of wild-type ubiquitin (lane 5 vs. lane 1 in Fig. 3E), perhaps because of a reduced assembly efficiency of the K48R ubiquitin. To test if NEDD4 directly ubiquitinates PPARγ, we performed PPARγ *in vitro* ubiquitination assay. Consistent with the *in vivo* ubiquitination data, we found that PPARγ was efficiently ubiquitinated by NEDD4 in a dose-dependent manner (Fig. 3F). Using wild-type (WT), K48-only (K48O), and K63-only (K63O) ubiquitin, we observed strong ubiquitination signals in each reaction, suggesting that NEDD4 catalyzes both K48 and K63 linkages (Fig. 3G).

### NEDD4 increases PPARγ protein stability

**Figure 4:**
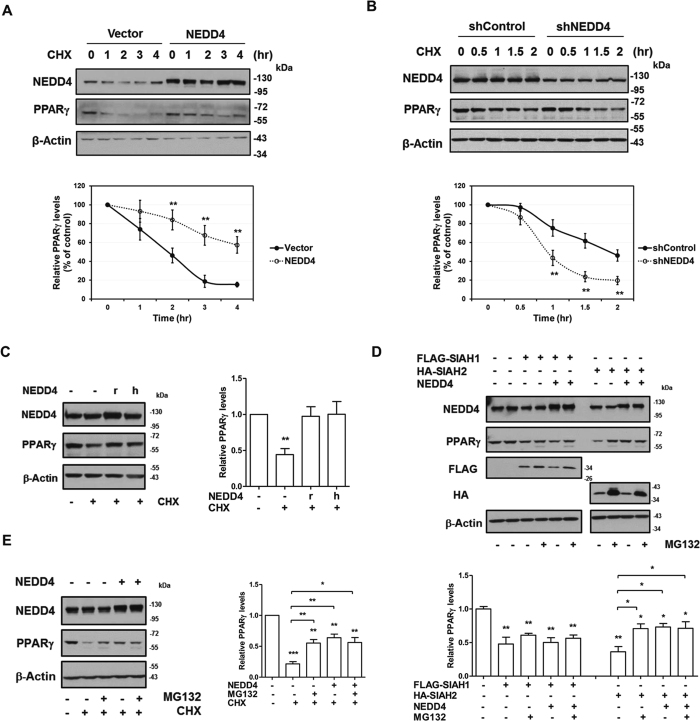
NEDD4 protects against rapid degradation of PPARγ. (**A**,**B**) NEDD4 regulates PPARγ protein half-life. Representative western blot image of PPARγ protein levels during cycloheximide-chase experiment. CHO cells stably expressing FLAG-tagged PPARγ2 were transfected with indicated plasmids. Two days after transfection, cells were treated with cycloheximide for the indicated times. Quantification of data is shown below. (**C**) The human (h) or rat (r) NEDD4 cDNAs were expressed in CHO cells stably expressing FLAG-tagged PPARγ2. Cycloheximide was added to the media 2 hr before cell harvesting. Quantification of data is on the right. (**D**,**E**) CHO cells stably expressing FLAG-tagged PPARγ2 were co-transfected with the indicated plasmids and treated with or without the indicated reagents. Cycloheximide and MG132 were added to the media 15 hr before cell harvesting. Cycloheximide was used at 20 μM. MG132 was used at 10 μM. Data represent mean ± SEM of 3–4 independent experiments; *P < 0.05, **P < 0.01, ***P < 0.001. CHX: cycloheximide. Full-length blots are presented in Supplementary Fig. S4.

PPARγ is a short-lived protein (t^1/2^ = 2 hr)^23^. Consistent with other studies, we found that PPARγ was rapidly degraded upon inhibition of de novo protein synthesis by cycloheximide. Pretreatment of the proteasomal inhibitor MG132, but not the lysosomal inhibitor chloroquine, reversed the degradation of PPARγ (Supplementary Fig. S1). Next, we measured the half-life of PPARγ by cycloheximide chase assay with overexpressed or downregulated NEDD4. Interestingly, overexpression of NEDD4 increased PPARγ half-life from 2 hr to >4 hr, while knockdown of NEDD4 destabilized PPARγ, with its half-life decreasing to 75 min (Fig. 4A,B).

The human and rat NEDD4 share 80% homology in the protein sequences. We compared the effect of NEDD4 from the two species on PPARγ protein degradation. Our results indicate that the overexpression of either human or rat NEDD4 protected against PPARγ degradation upon treatment with cycloheximide (Fig. 4C). Next, we tested whether NEDD4 was able to hinder the reduction of PPARγ caused by SIAH1 and SIAH2. MG132 treatment for 6 hr largely rescued SIAH2-mediated reduction, but, to our surprise, only slightly rescued SIAH1-mediated reduction. NEDD4 overexpression largely rescued SIAH2-mediated reduction of PPARγ to the level comparable with MG132 treatment, but had no effect on SIAH1-mediated reduction (Fig. 4D). NEDD4 overexpression combined with MG132 treatment did not further restore the decreased steady-state level of PPARγ mediated by SIAH2 or after addition of cycloheximide (Fig. 4D). NEDD4 overexpression combined with MG132 treatment did not further restore the decreased steady-state level of PPARγ by the addition of cycloheximide either (Fig. 4E). Our data suggest that NEDD4 blocks the PPARγ decrease by mechanisms similar to that of MG132, which is likely through inhibiting PPARγ proteasomal degradation.

### Knockdown of NEDD4 reduces PPARγ expression and inhibits adipogenic response in 3T3-L1 adipocytes

**Figure 5:**
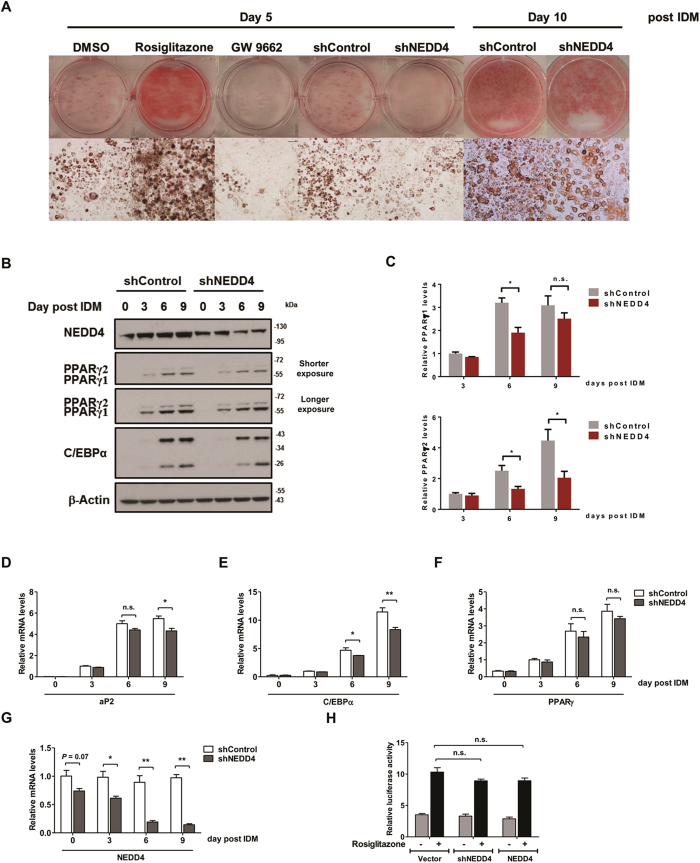
NEDD4 knockdown inhibits adipogenic response. (**A**) Photographs and micrographs of oil red O staining of the differentiated adipocytes. Either DMSO, 10 μM of rosiglitazone, or 10 μM of GW 9662 were added to the 3T3-L1 cells at the beginning of adipocyte differentiation and were replaced with culture media every 2 days. The 3T3-L1 cells were infected with AAV virus for expressing non-targeting shRNA control (shControl) or NEDD4-targeting shRNA (shNEDD4) 2 days before adipocyte differentiation. Photographs and micrographs were taken 5 days or 10 days after differentiation. Scale bar represents 40 μm. (**B**) Endogenous PPARγ and C/EBPα expression levels during 3T3-L1 adipocyte differentiation in the presence of AAV virus were measured by Western analysis. (**C**) Quantification of the endogenous PPARγ1 and PPARγ2 protein expression from panel B. (**D**–**G**) The mRNA levels of (**D**) aP2, (**E**) C/EBPα, (**F**) PPARγ, and (**G**) NEDD4 during 3T3-L1 adipocyte differentiation in the presence of AAV virus. (**H**) NEDD4 has no direct effect on the transcriptional activity of PPARγ. HEK293 cells were transiently co-transfected with 3 × PPRE-Luc, FLAG-tagged PPARγ2, *Renilla* luciferase control reporter vector pRL-SV40 and plasmids indicated under each column. After 32 hr, cells were treated with or without rosiglitazone (10 μM) for 16 hr prior to luciferase assay. Luciferase activity was normalized to *Renilla* activity as a control for transfection efficiency. IDM: 0.5 mM IBMX, 1 μM dexamethasone, and 1.5 μg/mL insulin. Data represent mean ± SEM of 3 independent experiments; *P < 0.05, **P < 0.01, n.s., no significance. Full-length blots are presented in Supplementary Fig. S4.

PPARγ is arguably the central regulator of adipogenesis. The stabilization of PPARγ by NEDD4 raises the question of whether NEDD4 regulates adipogenesis. To address this hypothesis, we employed adeno-associated virus (AAV)-delivery of specific shRNA to knockdown NEDD4 in 3T3-L1 cells, which are a well-established culture model for studying adipogenesis^24^. NEDD4 was reduced by >60% beyond day 5 post-differentiation (Fig. 5B,G). The knockdown of NEDD4 in 3T3-L1 cells significantly impeded the efficiency of the preadipocyte-to-adipocyte conversion as revealed by the oil red O staining at day 5 and 10 post-differentiation (Fig. 5A). To examine whether NEDD4 reduces PPARγ levels in 3T3-L1 adipocytes, we measured PPARγ expression these cells during the adipogenic conversion. Consistent with our hypothesis, PPARγ2 expression was significantly reduced beyond day 5 post-differentiation (*P* < 0.05). Significant reduction in PPARγ1 was only detected at day 6 post-differentiation (*P* < 0.05), possibly due to potential feedback and compensation effects on the γ1 which is more labile than γ2^13^ (Fig. 5B,C). Protein expression of C/EBPα, another adipogenic factor, was also reduced in the shNEDD4 AAV-infected 3T3-L1 adipocytes (Fig. 5B). The mRNA levels for *aP2* and *C*/*EBPα*, two PPARγ downstream target genes, were decreased by NEDD4 knockdown (Fig. 5D,E). Despite the decrease in PPARγ protein, its mRNA levels were not altered in comparison to those infected with non-targeting control shRNA AAV (Fig. 5F), confirming that NEDD4 regulates PPARγ at protein level. Of note, the expression of NEDD4 per se was not changed during adipocyte differentiation (Fig. 5B,G, Supplementary Fig. S2). We also examined whether NEDD4 influences PPARγ transcriptional activity. We performed PPARγ luciferase reporter assay. To our surprise, PPARγ transactivation by rosiglitazone was not altered by NEDD4 knockdown or NEDD4 overexpression (Fig. 5H). These data indicate that NEDD4 alters adipocyte differentiation primarily by regulating PPARγ abundance at the protein level, but not its transcriptional activation.

### NEDD4 is not essential for ligand-dependent degradation of PPARγ in 3T3-L1 adipocytes

**Figure 6:**
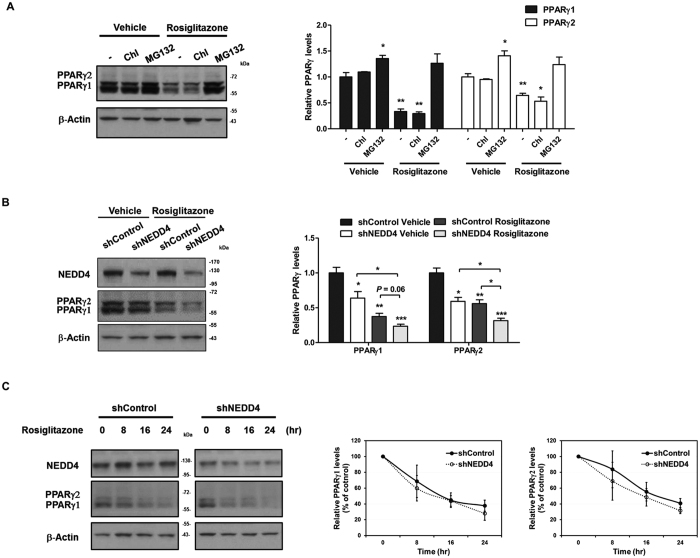
NEDD4 promotes PPARγ ubiquitination but is not required for ligand-dependent PPARγ degradation in 3T3-L1 adipocytes. (**A**) Representative western blot image of PPARγ expression in differentiated 3T3-L1 cells in the presence of ligand. The 3T3-L1 cells were differentiated for 8 days and treated with 5 μM of rosiglitazone for 16 hr with or without the pretreatment of lysosome inhibitor chloroquine (25 μM) or proteasome inhibitor MG132 (10 μM). Quantification figure is on the right. (**B**) Effect of NEDD4 knockdown on PPARγ expression in differentiated 3T3-L1 cells in the presence or absence of ligand. The 3T3-L1 cells infected with shControl or shNEDD4 AAV were differentiated for 6 days and treated with or without 5 μM of rosiglitazone for 16 hr. Representative western blot image is shown and its quantification figure is shown below. (**C**) NEDD4 knockdown reduces PPARγ ubiquitination in 3T3-L1 adipocytes in the presence or absence of ligand. The 3T3-L1 cells infected with shControl or shNEDD4 AAV were differentiated for 6 days and treated with or without 5 μM of rosiglitazone for 16 hr. MG132 (10 μM) was added to the media 6 hr before harvesting the cells. Arrows indicate NEDD4, PPARγ1 and γ2. (**D**) Representative western blot image of time-dependent expression changes of PPARγ in AAV infected 3T3-L1. The 3T3-L1 cells infected with shControl or shNEDD4 AAV were differentiated for 2–3 days and treated with or without 5 μM of rosiglitazone for 8–24 hr. Quantification figures are shown on the right. Data represent mean ± SEM of 3 independent experiments; *P < 0.05, **P < 0.01, ***P < 0.001. Full-length blots are presented in Supplementary Fig. S4.

Consistent with previous findings^12^,^20^, treatment of differentiated 3T3-L1 cells with the PPARγ agonist rosiglitazone induced both PPARγ1 and γ2 protein downregulation which was blocked by pretreatment of the proteasome inhibitor MG132 (Fig. 6A). To test if NEDD4 has an effect on ligand-induced degradation of PPARγ, we knocked down NEDD4 expression in 3T3-L1 cells and incubated the cells with or without rosiglitazone. Rosiglitazone induced significant downregulation in PPARγ1 and γ2 protein. Quantified results showed that the γ1and γ2 protein levels were significantly decreased after rosiglitazone treatment. Knockdown of NEDD4 further downregulated the expression of γ1 and γ2. However, the percentages of decrease were similar to those in shControl AAV infected cells (Fig. 6B), suggesting that NEDD4 is not essential for ligand-dependent degradation of PPARγ. We further examined the levels of ubiquitinated endogenous PPARγ in AAV infected 3T3-L1 cells with or without rosiglitazone treatment. As the cells treated with rosiglitazone were associated with lower total PPARγ protein levels, the amount of protein immunoprecipitated down from ligand-treated cells was also less. Our results showed that shNEDD4 AAV infected cells contained lower levels of ubiquitinated PPARγ compared with that in shControl AAV infected cells in the presence or absence of ligand (Fig. 6C). The expression of both PPARγ isoforms during time-dependent treatment of rosiglitazone in shNEDD4 AAV infected cells was not different from the control (Fig. 6D). We conclude that NEDD4 promotes ubiquitination of endogenous PPARγ in differentiated 3T3-L1 cells but is not essential in the ligand-dependent decrease of PPARγ protein.

### NEDD4 positively correlates with PPARγ protein levels in obese adipose tissue

**Figure 7:**
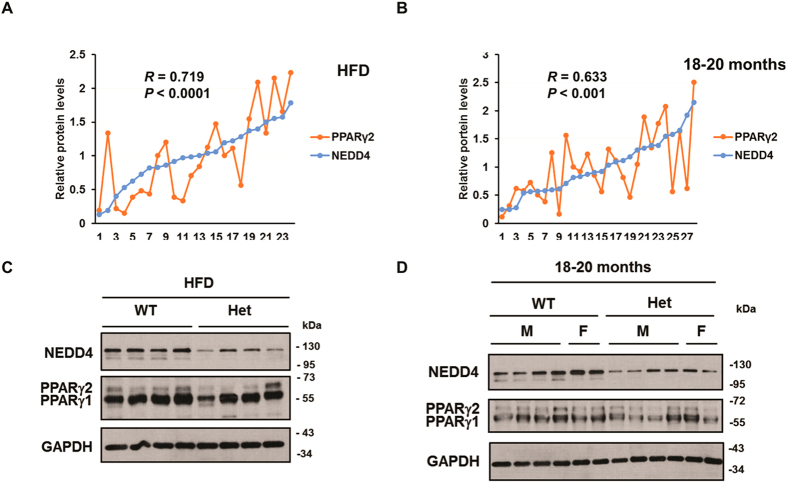
Positive correlation between NEDD4 and PPARγ levels in obese adipose tissue. (**A**,**B**) Pearson’s R correlation coefficient between steady-state NEDD4 and PPARγ2 protein abundance in epididymal fat in 24 male HFD-fed wild-type (WT) and *Nedd4*^+/−^ (Het) mice (WT: n = 12; Het: n = 12) or 28 aged (18–20 months) WT and Het mice (WT: n = 14, 12 males and 2 females; Het: n = 14, 12 males and 2 females). (**C**,**D**) Representative western blot image of NEDD4 and PPARγ expression in epididymal fat in HFD-fed or aged WT and Het mice. The HFD-fed mice at 6-week of age were fed a HFD (TD.06414, Teklad, Harlan Laboratories), containing 60% calories from fat, for 16 weeks. Samples were arranged in increasing order of NEDD4 expression. Full-length blots are presented in Supplementary Fig. S4.

To study the correlation between NEDD4 and PPARγ expression in obese adipose tissue, we analyzed NEDD4 and PPARγ protein expression in mixed epididymal fat tissue samples from wild-type and *Nedd4* heterozygous (*Nedd4*^+/−^) mice fed a HFD or from aged wild-type and *Nedd4*^+/−^ mice. The samples were arranged in increasing order of NEDD4 expression. We then calculated the Pearson’s R correlation coefficients between NEDD4, PPARγ1 and γ2 protein levels. A significant positive correlation was shown between NEDD4 and PPARγ2 in the HFD group (*R* = 0.719, *P* < 0.0001; Fig. 7A and C) and the aged group (*R* = 0.633, *P* < 0.001; Fig. 7B and D). A statistically significant positive correlation was observed between PPARγ1 and γ2 as expected (HFD: *R* = 0.535, *P* < 0.01; aged: *R* = 0.443, *P* < 0.05; Supplementary Fig. S3A,B). Surprisingly, there was no statistical significant correlation between NEDD4 and PPARγ1 in these samples (Supplementary Fig. S3C,D).

## Discussion

We have shown here that the ubiquitin ligase NEDD4 stabilizes PPARγ, promoting adipogenesis in 3T3-L1 cells. The evidence supporting this conclusion is as follows. First, NEDD4 interacts with and ubiquitinates PPARγ. Second, up- or down-regulation of NEDD4 prolongs or shortens the PPARγ protein half-life respectively. Third, knockdown of NEDD4 in 3T3-L1 cells reduces PPARγ expression and blocks adipocyte differentiation. Finally, the protein levels of NEDD4 and PPARγ are positively correlated in obese adipose tissue.

PPARγ plays a central role in adipocyte differentiation. Increasing attention has been paid to PPARγ posttranslational modification, which includes phosphorylation, sumoylation and ubiquitination^23^. Despite the fact that PPARγ contains a typical PPxY binding motif for the WW domains of NEDD4, we did not observe interaction through this motif, as the amount of NEDD4 in the complex with PPxY mutant PPARγ was the same found in complex with wild-type PPARγ. Previous reports showed that WW-domain containing E3 ligases can be recruited by substrates in a PPxY-independent manner^25^,^26^,^27^. Our data indicated that NEDD4 binds the hinge/LBD domain of PPARγ, and PPARγ missing the hinge and LBD domains was cleaved into smaller fragments, suggesting that these two domains are critical for the stability of PPARγ. The hinge region has been reported to regulate the subcellular distribution and trafficking of many nuclear receptors^28^. This region has also been shown to serve as an interaction site for modulators of nuclear receptors by numerous studies. Such a function has been demonstrated for androgen receptor^29^,^30^,^31^, estrogen receptor^32^,^33^,^34^,^35^,^36^, glucocorticoid receptor^37^,^38^, progesterone receptor^39^, PPARα^39^, and PPARγ^40^,^41^,^42^. The LBD has been more extensively studied in structure and function. The activation function 2 (AF-2) region within LBD can recruit co-activators via the amino acid LxxLL motif^43^. In line with these published reports, our findings add to the evidence that hinge and LBD domains of nuclear receptors are critical for nuclear receptor modulator and co-regulator recruitment.

Our data suggest that NEDD4 facilitates both K48 and K63-linked polyubiquitination of PPARγ *in vitro*, although the exact ubiquitin-chain type(s) formed *in vivo* may be influenced by other binding factors or different E2-E3 interactions. For example, (1) other binding factors may position PPARγ differently in E3-substrate complex. In this case, lysine preference in a given ubiquitination reaction would be affected. Alternatively, (2) the polyubiquitin chain is initially built by one or more E2s on the HECT cysteine residue. In this respect, the linkage specificity would be determined by the E2. NEDD4 has been shown to preferentially synthesize K63-linked ubiquitin chains *in vivo* and *in vitro* in numerous reports^44^. In our case, ubiquitin proteasome pathway is the major pathway for PPARγ degradation. We did not observe degradation of PPARγ when overexpressing NEDD4, suggesting that the ubiquitin chains added to PPARγ by NEDD4 fails to target PPARγ to the proteasomes. Therefore, it is less likely that K48-linked polyubiquitination is the major chain type formed by NEDD4 in PPARγ in cells. It is possible that NEDD4 preferentially mediates non-K48-linked polyubiquitination (*eg*. K63 linkage) *in vivo* which competes with the K48-linked polyubiquitination in PPARγ and therefore protects PPARγ from proteasomal degradation. It is also possible that a certain type of uncharacterized ubiquitin chain masks the recognition of PPARγ by proteasomes. Despite the fact that NEDD4 ubiquitinates PPARγ, we cannot rule out the possibility that direct binding of NEDD4 to PPARγ also contributes to the stabilization of PPARγ protein. Future studies could consider replacing the active-site cysteine residue in NEDD4 HECT domain with serine residue by genome editing, and see if PPARγ stability is affected. However, doing so might affect other substrates of NEDD4 as well which may exert feedback regulations on PPARγ.

Although differential posttranslational regulation of the two PPARγ isoforms has not been previously demonstrated, functional differences between PPARγ1 and γ2 have been shown^9^. That the PPARγ isoforms could be differentially regulated is not surprising. In the case of our study, NEDD4 affects PPARγ1 and γ2 differently. PPARγ1 expression decreases when NEDD4 is knocked down but to a lesser extent than the PPARγ2 expression. Previously published data showed that there are no ubiquitination sites in the A/B domain. Neither the PPARγ1 nor γ2A/B domain are modified by ubiquitin. However, those domains do seem to influence stability and proteasome degradation independent of ubiquitin modification^21^. The turnover rate of PPARγ1 and γ2 are also different – the half-life of γ1 is shorter than γ2^20^, so the N-terminal extension has an effect on stability whether or not a ligand is bound in the LBD. There is a previous study showing that the A/B domain can influence ligand binding through interdomain communication^45^, so it is possible that other posttranslational modifications of the γ2A/B domain affect NEDD4-mediated ubiquitination and the stability of PPARγ, especially considering the fact that the N-terminal 30 amino acids of the PPARγ2 contain several serine and threonine residues.

Polyubiquitinated proteins are normally degraded by the 26S proteasome^46^. It is easy to postulate that knockdown of PPAR-γ E3 ligase reduces ubiquitination of PPAR-γ and thus there is less PPAR-γ degradation and more transactivation leading to increased adipogenesis. In fact, the relationships between PPARγ ubiquitination, degradation and transcriptional activation are not always straight-forward. The E3 ligase SIAH2 has been shown to ubiquitinate PPARγ for degradation and to decrease its activity^16^. Depletion of SIAH2 in 3T3-L1 preadipocytes, however, prohibits adipocyte differentiation^16^. Another example is the E3 ligase TRIM23 which has recently been shown to ubiquitinate PPARγ, not for degradation, but rather, stabilization^17^. TRIM23 knockdown inhibits adipogenesis, but it does not appears to affect PPARγ transcriptional activity^17^. Similar to TRIM23’s effects, we found that NEDD4 stabilizes PPARγ. Using a reporter gene assay, we demonstrated that the transcriptional activity of PPARγ is not altered by either NEDD4 overexpression or knockdown. Other substrates and signaling pathways that are regulated by NEDD4 may account for this puzzling outcome. It has been reported that NEDD4 can ubiquitinate tumor suppressor phosphatase and tensin homolog (PTEN), protein kinase B (PKB/AKT) and insulin signaling molecules^47^,^48^,^49^, all of which are involved in lipid metabolism. However, the regulation of PPARγ activity by these insulin signaling molecules in adipocytes has yet to be clarified. The final outcome of PPARγ activation, therefore, may be a balance between different NEDD4 substrates’ regulation on PPARγ activity. These observations reveal differential roles for different E3 ligases in adipogenesis and PPARγ regulation. Because most mechanistic studies were performed with ectopically overexpressed PPARγ, PPARγ may stand out among other substrate candidates in this scenario. When performing adipogenesis or animal studies, other substrate(s) may be more preferentially regulated than the endogenous PPARγ by the E3 ligases. Therefore, whether the phenotypes in adipocytes are direct effects through PPARγ per se or mixed effects involved other pro-/anti-adipogenic molecules are unclear. Moreover, a remaining unsolved question is what are the functionally important regions and residues within PPARγ that are regulated by NEDD4. Delineating such regions may advance in the development of novel pharmacological agents for selective PPARγ stability modulators as alternatives to PPARγ agonists.

## Methods

### Immunoblot Analysis

Cells or tissues were lysed in radioimmunoprecipitation (RIPA) buffer supplemented with protease and phosphatase inhibitors. Cell debris in lysates was removed by centrifugation at maximum speed at 4 °C. Protein concentrations were determined by the bicinchoninic acid (BCA) method (Thermo Fisher Scientific, Waltham, MA). Sample were re-suspended in sodium dodecyl sulfate (SDS) sample buffer and denatured in boiling water for 5 min, then electrophoresed on a Novex 4–20% Tris-Glycine gel (Invitrogen, Carlsbad, CA). After transferring the proteins to a polyvinylidene difluoride (PVDF) membrane (Millipore, Billerica, MA), the membrane was blocked at room temperature in 1 × Tris-buffered saline plus 0.05% Tween-20 (TBST) containing 5% non-fat dry milk for 1 hr. After blocking, the membrane was incubated overnight at 4 °C in primary antibody diluted in TBST/5% BSA/0.02% NaN_3_. On the second day, the membrane was washed thoroughly with TBST and incubated in secondary horseradish peroxidase (HRP) linked antibody (GE Healthcare Life Sciences, Pittsburgh, PA). The blot was washed three times with TBST followed by incubation with enhanced chemiluminescence (ECL) for signal development. For data presented in Fig. 7, samples were run on different gels. Each gel had an equal number of wild-type samples and *Nedd4*^+/−^ samples. The intensity of protein NEDD4 and β-Actin bands was quantified by ImageJ. Then each of the densitometry values of NEDD4 was normalized to value of β-Actin of the same sample. The NEDD4/β-Actin ratio values of all samples were averaged from each blot to obtain a ratio value specific to each blot. The values of NEDD4/β-Actin on other blots were then adjusted to the first blot by the average ratio values of each blot. An average NEDD4/β-Actin value (X) for all samples was calculated. NEDD4/β-Actin ratio value of each sample was normalized to X, and was arranged in increasing order of NEDD4 expression. Same quantification method was applied for PPARγ1 and γ2.

### Cell culture, Plasmids and Transfection

The HEK293 cells were cultured in high glucose Dulbecco’s Modified Eagle Medium (DMEM) supplemented with 10% fetal bovine serum (FBS). The Chinese hamster ovary (CHO) cells stably expressing FLAG-tagged PPARγ2 were maintained in DMEM plus 10% FBS and 200 μg/mL of G418. Media was replaced every 2–3 days. Cells were incubated in 95% O_2_ and 5% CO_2_ at 37 °C. The pcDNA3.1-PPARγ2-FLAG (# 8895), pRK5-HA-Ubiquitin-WT (# 17608), and pRK5-HA-Ubiquitin-K48R (# 17604) plasmids were obtained from Addgene (Cambridge, MA). The PPARγ2 PPYA, AAYA, and ∆PPYY mutants were generated by the QuikChange II Site-Directed Mutagenesis Kit (Agilent Technologies, Santa Clara, CA). The FLAG-tagged PPARγ2 deletion mutants that were missing the hinge domain (∆Hinge, without amino acids 206–280), LBD (∆LBD, without amino acids 281–505), or both hinge and LBD domains (∆∆, without amino acids 206–505) were created by PCR using the pcDNA3.1-PPARγ2-FLAG as a template. All constructs were sequenced to confirm mutations and deletions. The pRc-CMV-T7-NEDD4 and pRc-CMV-T7-NEDD4 CS plasmids containing wild-type or catalytically inactive (bearing a C to S mutation at the HECT domain) rat NEDD4 were kind gifts from Dr. Daniela Rotin (The Hospital for Sick Children, Toronto, ON, Canada). The pcDNA3.1.1-NEDD4-1-HA plasmid containing a human NEDD4 cDNA was kindly provided by Dr. Xuejun Jiang from Memorial Sloan-Kettering Cancer Center (New York, NY, USA). The pcDNA3.1-FLAG-SIAH1 plasmid was generously given by Dr. Ze’ev Ronai (The Burnham Institute, La Jolla, CA, USA). The pcDNA3.1.1-HA-SIAH2, GAL4DBD-HA-AF-1, GAL4DBD-HA-DBD, GAL4DBD-HA-Hinge, and GAL4DBD-HA-LBD plasmids were provided by Dr. Elizabeth Floyd (Pennington Biomedical Research Center, Baton Rouge, LA, USA). Cells were transfected with Lipofectamine 2000 (Invitrogen, Carlsbad, CA) following the manufacturer’s instructions. After 48 hr, the cells were harvested and lysed in RIPA buffer for immunoblot analysis.

### Adeno-associated Virus (AAV)-mediated Knockdown

The pAAV2.1.CMV.EGFP-U6. shRNA plasmid vector (a kind gift from Dr. Tonia Rex at Vanderbilt University, Nashville, TN, USA) was used to generate AAV virus. Of the three different short hairpin RNA (shRNA) constructs tested, the one showing most effective downregulation of mouse NEDD4 protein and mRNA (>50%) was chosen for subsequent studies. The targeting sequence was 5′-TGGCGATTTGTGAACCGTA-3′. The non-targeting control sequence was 5′-CAACAAGATGAAGAGCACCAA-3′. Virus production was performed by the Gene Transfer Vector Core at the University of Iowa.

### Differentiation of 3T3-L1 Cells and Oil Red O Staining

The 3T3-L1 preadipocytes were cultured in high glucose DMEM supplemented with 10% bovine calf serum. Cells were split 1:10 every 2 days to prevent reaching confluency. Cells were incubated in 95% O_2_ and 5% CO_2_ at 37 °C. For adipocyte differentiation, the preadipocytes were allowed to grow to confluency in 10% bovine calf serum. Two days post confluency, cells were switched to DMEM supplemented with 10% fetal bovine serum (FBS) and stimulated with a differentiation cocktail (IDM) containing 0.5 mM 3-isobutyl-1-methylxanthine (IBMX), 1 μM dexamethasone, and 1.5 μg/mL insulin. Two days after IDM induction, cells were fed with DMEM plus 10% FBS and 1.5 μg/mL insulin. Three days later, cells were cultured in DMEM plus 10% FBS and media was replaced every 2–3 days until full differentiation was achieved. For oil red O staining, a stock oil red O solution was prepared by adding 150 mg of oil Red O powder to 50 mL of isopropanol. Then, 3 parts of oil Red O stock solution were mixed with 2 parts distilled water and incubated for 10 min at room temperature. The oil red O working solution was filtered through Whatman filter paper. Differentiated 3T3-L1 adipocytes cultured on 6-well dishes were first fixed in 10% formalin for 30–60 min. Cells were then briefly rinsed with 3 times with distilled water and incubated in 60% isopropanol for 5 min. Freshly prepared oil red O working solution was added to the cells for 5 min. Cultures were rinsed with room temperature tap water until the water rinses were clear. The stained lipid droplets were photographed with a digital camera. Bright-field images were captured with the IX50 inverted system microscope (Olympus corporation of the Americas) using magnification of ×40. A non-stained area was chosen to set the white balance.

### Co-Immunoprecipitation

Cells were collected in a solution containing 25 mM Tris-HCl, 150 mM NaCl, and 1% NP-40 supplemented with protease and phosphatase inhibitors. Lysates were passed 5 times through a 25 G syringe to increase protein extraction. The lysates were centrifuged to obtain soluble proteins. After determining the protein concentrations, ~500 μg of total protein was used for a co-immunoprecipitation (co-IP) assay. Then, 1 μg of antibody or IgG, and 30 μl of protein A/G beads (Santa Cruz Biotechnology, Dallas, TX) were added to each sample for overnight incubation with gentle shaking at 4 °C. On the second day, beads were centrifuged at 5,000 rpm for 2 min, washed three times with the co-IP buffer, and finally resuspended in 1 × SDS sample buffer and denatured in boiling water for 5 min. Samples were centrifuged again at maximum speed and the resulting supernatant was loaded directly on a Tris-Glycine gel or stored at −80 °C until use.

### *In Vivo* Ubiquitination Assay

HEK293 cells were transfected with combinations of plasmids encoding PPARγ2-FLAG, NEDD4, and ubiquitin-WT or ubiquitin-K48R. After 36 hr, cells were treated with 10 μM MG132 for 12 hr. The cells were harvested and lysed in 25 mM Tris-HCl, 150 mM NaCl, 1% NP-40, 0.1% SDS, and 0.5% sodium deoxycholate supplemented with protease and phosphatase inhibitors. At least 1 mg of total protein was used for the *in vivo* ubiquitination assay. Then, 2 μg of antibody and 40 μl of protein A/G beads (Santa Cruz Biotechnology, Dallas, TX) were added to each sample for overnight incubation with gentle shaking at 4 °C. The following steps were similar to those described in co-IP.

### *In Vitro* Ubiquitination Assay

Two sets of *in vitro* ubiquitination reactions were implemented. First for the dose-dependent PPARγ2 ubiquitination by NEDD4 and second for NEDD4 induced PPARγ2 ubiquitination with wild-type (WT)-Ub, K48O-Ub and K63O-Ub. For both the reaction assays, the premixture constituted of: 2 mM DTT, 5 mM MgCl_2_, 40 mM Tris-HCl (pH 7.5), 5 mM ATP, 0.70 M Sucrose, 8 mM CHAPS, 40 nM HM-E1, 350 nM E2-UbcH5c and 400 nM of recombinant NEDD4 protein. PPARγ2 dose-dependent ubiquitination by NEDD4 reaction mixture received 10 μM WT-Ub, and varying concentrations of PPARγ2 protein ranging from 25 ng to 200 ng while the second reaction mixture for PPARγ2 ubiquitination with different ubiquitin received 50 ng and 100 ng of PPARγ2 protein with either 20 μM WT-Ub, 100 μM K48O-Ub or 100 μM K63O-Ub. The reactions were initiated by incubation of the mixtures at 30 °C water bath for one hour then terminated by adding SDS sample buffer, followed by SDS-PAGE and Western Blot analysis for anti-Ub and PPARγ2.

### Quantitative Real-time PCR

For the 3T3-L1 adipocyte samples, 500 μl Trizol reagent (Invitrogen, Carlsbad, CA) was applied per well in a 12-well plate; cells were lysed by scraping. For adipose tissue samples, 1 ml Trizol reagent was applied per 50–100 mg of tissue; cells were lysed using a homogenizer. Total RNA was isolated as described in the manufacturer’s instructions. Primers used in this study included: *PPARγ*, forward 5′-GAAAGACAACGGACAAATCACC-3′, reverse 5′-GGGGGTGATATGTTTGAACTTG-3′; *C*/*EBPα*, forward 5′-GAGCAAAAATGTGCCTTGATATT-3′, reverse 5′-TGCACCCTTCATTTTTCTCAC-3′; *aP2*, forward 5′-GGATGGAAAGTCGACCACAA-3′, reverse 5′-TGGAAGTCACGCCTTTCATA-3′; *NEDD4*, forward 5′-ACGTGCTGTTCACTGCTGAT-3′, reverse 5′-TCACAACTCGTGTGTCATCG-3′; *GAPDH*, forward 5′-GCAAATTCAACGGCACAG-3′, reverse 5′-CTCGCTCCTGGAAGATGG-3′.

### Dual Luciferase Reporter Assay

HEK293 cells of relatively equal numbers were plated on 24-well plates. On the second day, seeded cells were transiently co-transfected with 250 ng 3 × PPRE-Luc, 250 ng PPARγ2-FLAG, 10 ng Renilla luciferase control reporter vector pRL-SV40 and 250 ng of other plasmids using Lipofectamine 2000 transfection reagent (Invitrogen, Carlsbad, CA). At 32 hr after the transfection, cells were treated with rosiglitazone (10 μM) for 16 hr. The luciferase assay was performed by a dual luciferase assay system (Promega, Madison, WI) according to the manufacturer’s instructions. The luciferase activity was adjusted by the Renilla luciferase activity to remove the variation caused by transfection efficiency.

### Chemicals and Antibodies

Chloroquine, MG132, cycloheximide, rosiglitazone, GW 9662, isobutylmethylxanthine (IBMX), dexamethasone, insulin and oil red O were purchased from Sigma-Aldrich (St Louis, MO). The anti-NEDD4 rabbit polyclonal antibody (catalog# 07-049) was purchased from Millipore (Billerica, MA). The anti-C/EBPα rabbit polyclonal antibody (catalog# 2295) was obtained from Cell Signaling Technology (Danvers, MA), and the anti-PPARγ (E-8) mouse monoclonal antibody and the anti-PPARγ (H-100) rabbit polyclonal antibody were purchased from Santa Cruz Biotechnology (Dallas, TX). The anti-PPARγ rabbit monoclonal antibody (catalog# MA5-15003) was from Thermo Fisher Scientific (Waltham, MA). The anti-HA.11 epitope tag mouse monoclonal antibody (previously Covance catalog# MMS-101P) was purchased from BioLegend (San Diego, CA). The anti-FLAG epitope tag (M2, catalog# F1804), the anti-GAPDH (catalog# G8795) and the anti-β-actin (catalog# A2228) mouse monoclonal antibodies were from Sigma-Aldrich (St Louis, MO). The mono- and polyubiquitinylated conjugates monoclonal antibody (FK2) was purchased from Enzo Life Sciences (Farmingdale, NY). The recombinant human PPARγ2 with His and GST tag (catalog# 501331) was purchased from NovoPro (Shanghai, China). The anti-Ub antibody was purchased from BD Biosciences (San Jose, CA).

### Statistical Analysis

All data represent mean ± SEM. Two-tailed, paired Student’s *t*-test was used to analyze the statistical significance of data. Statistical significance was determined by *P* value of less than 0.05 (*P* < 0.05).

## Additional Information

**How to cite this article**: Li, J. J. *et al*. Ubiquitin Ligase NEDD4 Regulates PPARγ Stability and Adipocyte Differentiation in 3T3-L1 Cells. *Sci. Rep.* **6**, 38550; doi: 10.1038/srep38550 (2016).

**Publisher's note:** Springer Nature remains neutral with regard to jurisdictional claims in published maps and institutional affiliations.

## Supplementary Material

Supplementary Information
