# Supplementary material for: Ubiquitin Ligase NEDD4 Regulates PPARγ Stability and Adipocyte Differentiation in 3T3-L1 Cells
Source: Sci Rep. 2016 Dec 5;6:38550. doi: 10.1038/srep38550 (PMC5137149; doi:10.1038/srep38550)
Supplement: Supplementary Information [file srep38550-s1.pdf]

Supplementary Information

Ubiquitin Ligase NEDD4 Regulates PPARγ Stability and Adipocyte Differentiation in 3T3-L1 Cells

Jing Jing Li, Ruishan Wang, Rati Lama, Xinjiang Wang, Z. Elizabeth Floyd, Edwards A. Park,

Francesca-Fang Liao

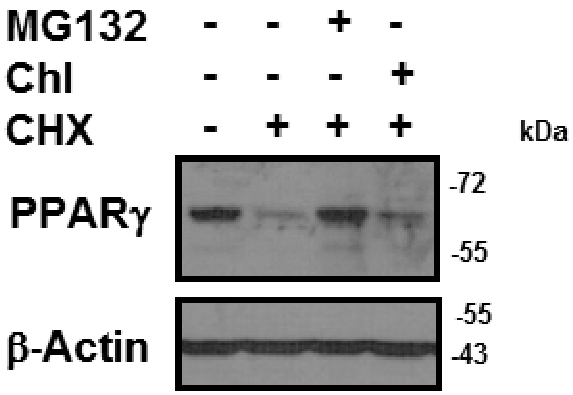

Supplementary Figure S1. PPARγ is susceptible to proteasomal-dependent degradation. HEK293 cells transiently expressing FLAG-tagged PPARγ2 were treated with or without 20 µM cycloheximide 15 hr before cell harvesting. MG132 (20 µM) or chloroquine (50 µM) were added to the cells 1 hr prior to the addition of cycloheximide. CHX: cycloheximide.

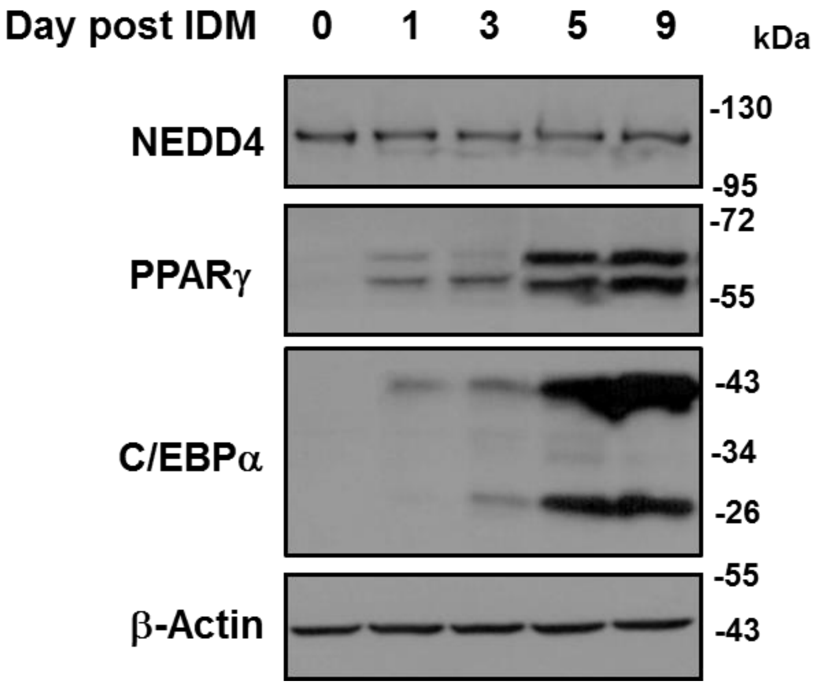

Supplementary Figure S2. Unaltered NEDD4 expression during adipocyte differentiation. After 1, 3, 5, and 9 days of differentiation, 3T3-L1 cells were harvested. Total cell lysates were immunoblotted for indicated proteins.

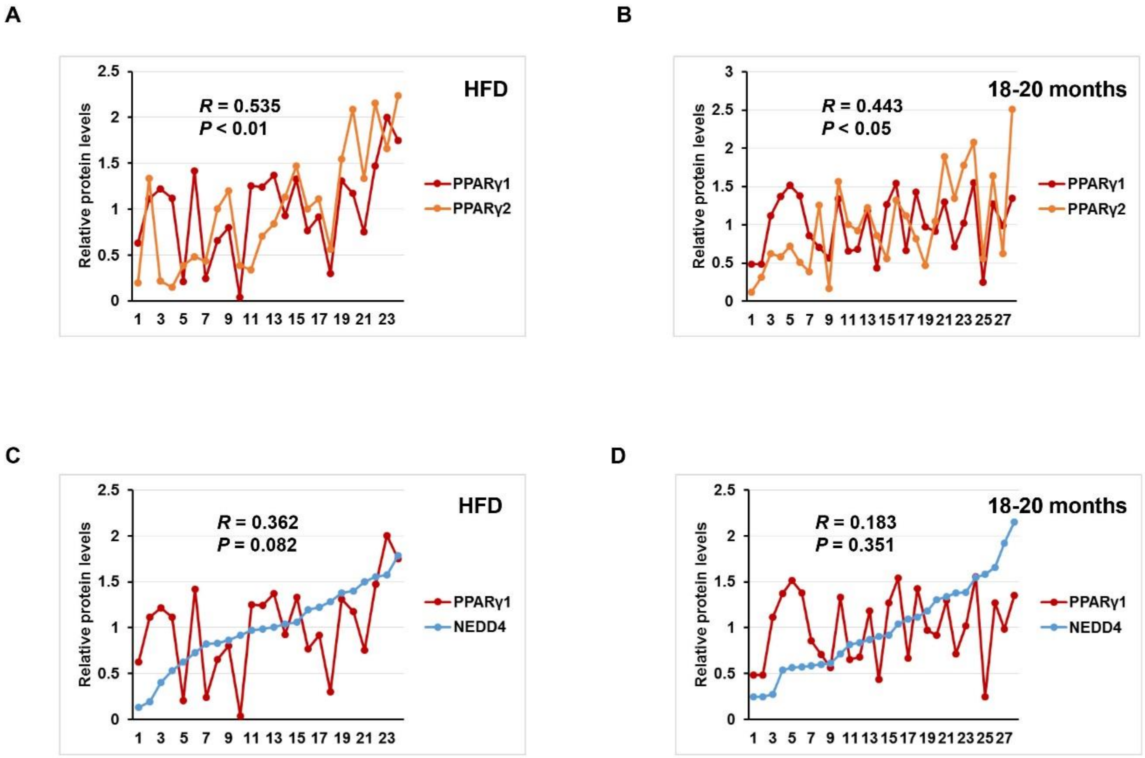

Supplementary Figure S3. Protein abundance correlation in adipose tissue.
(A and B) Significant positive correlation between PPARγ1 and PPARγ2 in epididymal fat in (A) 24 male HFD-fed WT and Het mice (WT: n=12; Het: n=12) or (B) 28 aged (18-20 months) WT and Het mice (WT: n=14, 12 males and 2 females; Het: n=14, 12 males and 2 females). (C and D) Pearson's R correlation coefficient between steady-state NEDD4 and PPARγ1 protein abundance in epididymal fat in (C) 24 male HFD-fed WT and Het mice (WT: n=12; Het: n=12) or (D) 28 aged (18-20 months) WT and Het mice (WT: n=14, 12 males and 2 females; Het: n=14, 12 males and 2 females). The HFD-fed mice at 6-week of age were fed a HFD (TD.06414, Teklad, Harlan Laboratories), containing 60% calories from fat, for 16 weeks. Samples were arranged in increasing order of NEDD4 expression.

Fig 1A

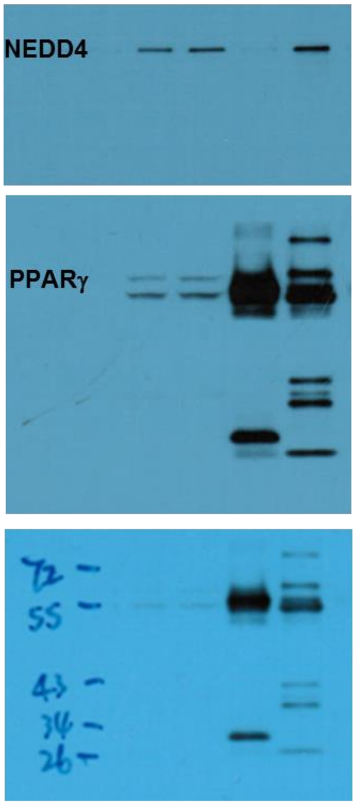

Fig 1B

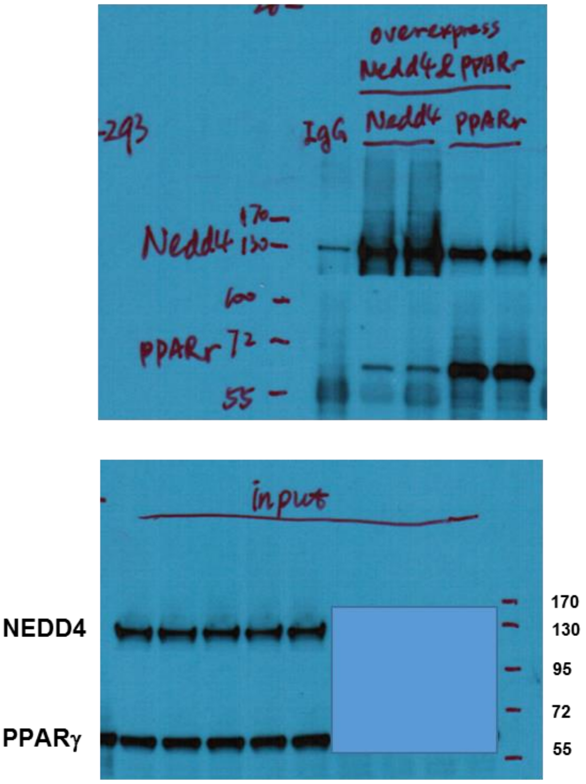

Fig 1C

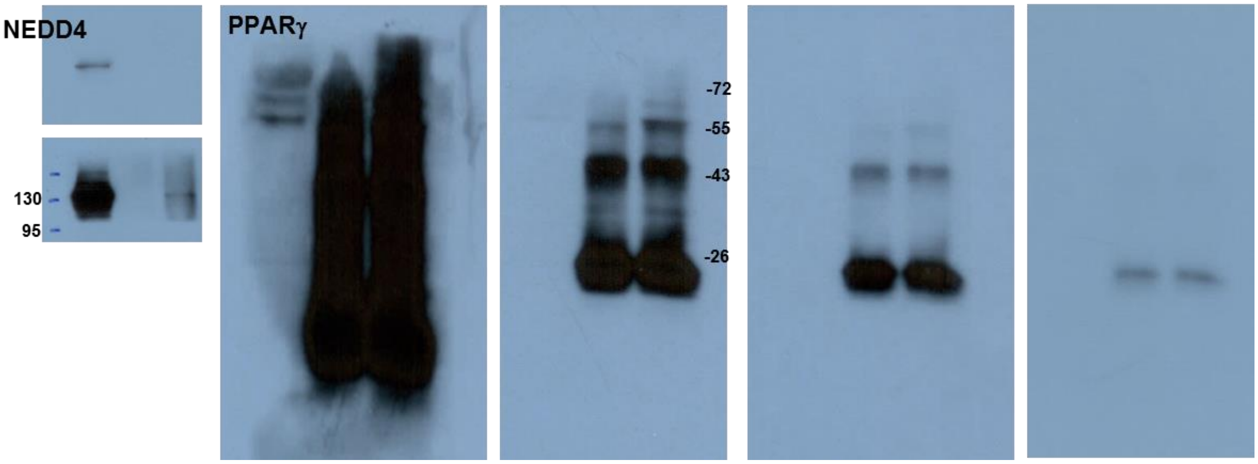

Supplementary Figure S4. Full-length western blot images of Figures 1-7.

Fig 2B

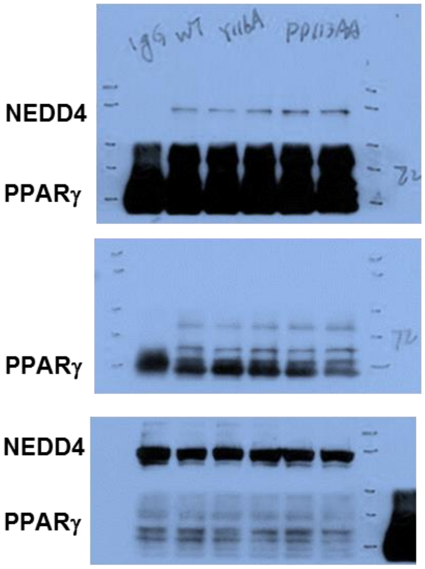

Fig 2C

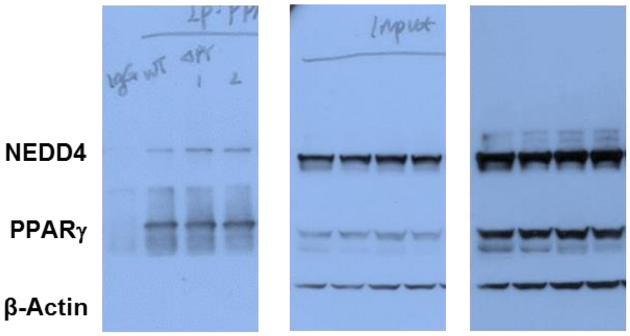

Fig 2D

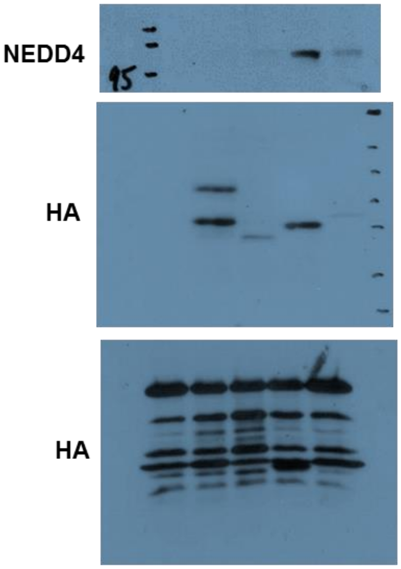

Fig 2E

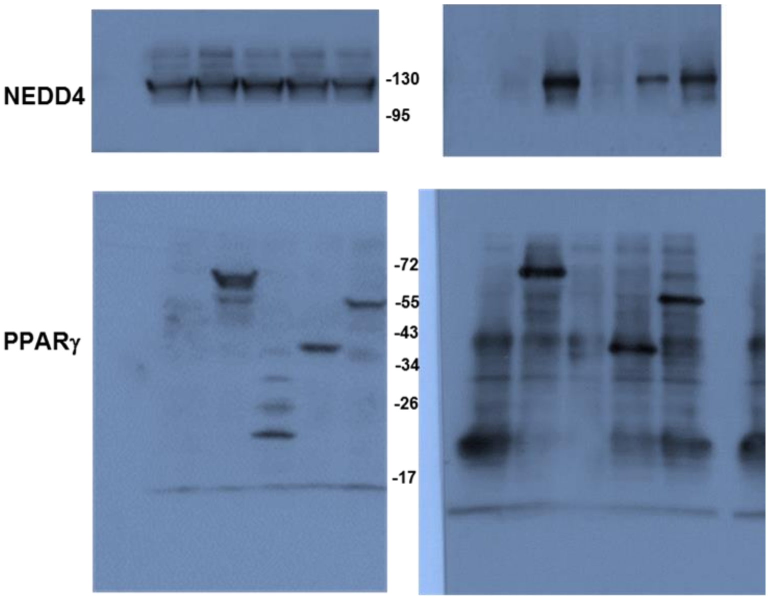

Supplementary Figure S4 continued

Fig 3A

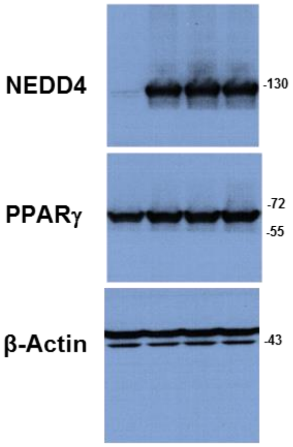

Fig 3B

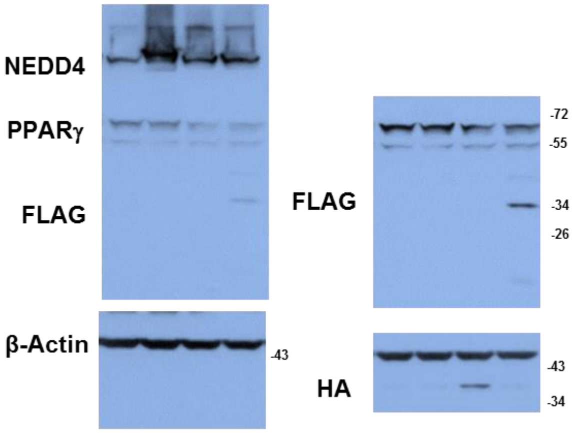

Fig 3C

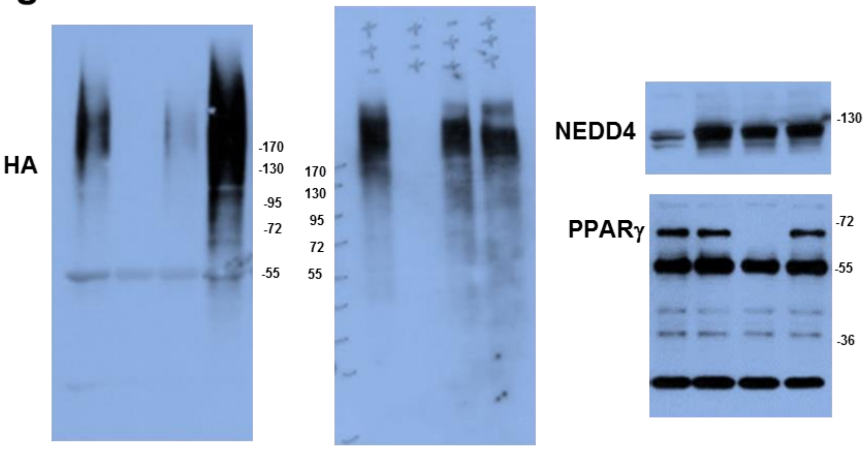

Fig 3D

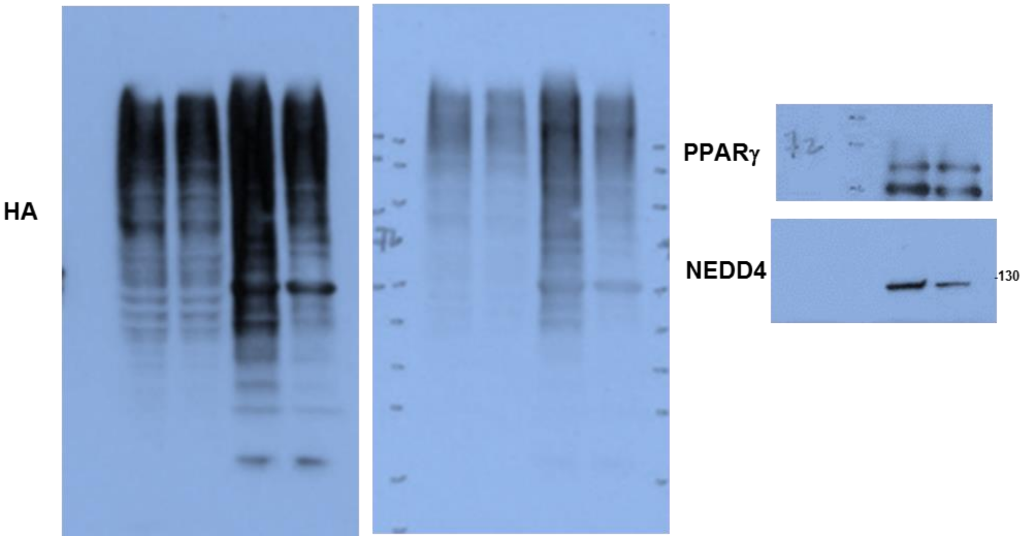

Supplementary Figure S4 continued

Fig 3E

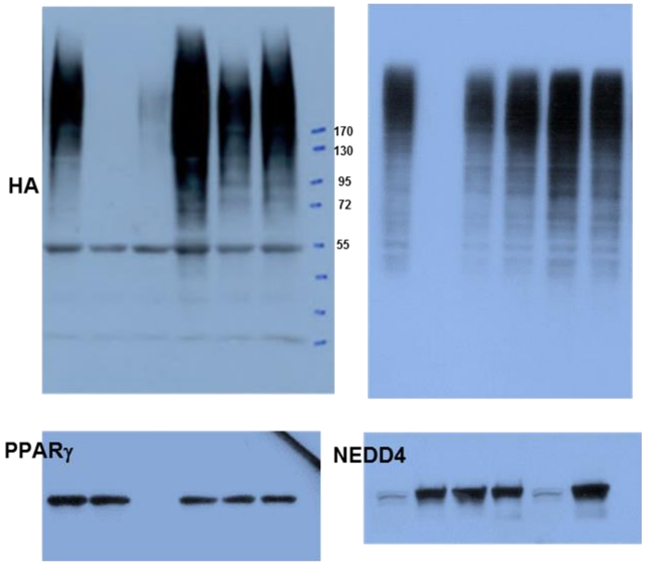

Fig 3F

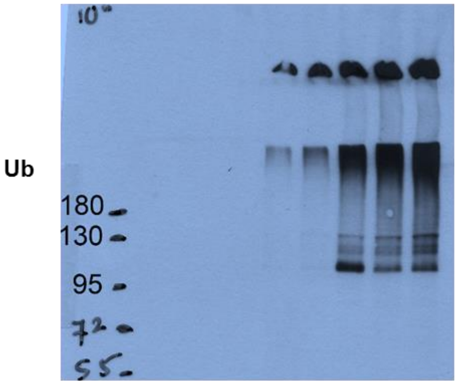

Fig 3G

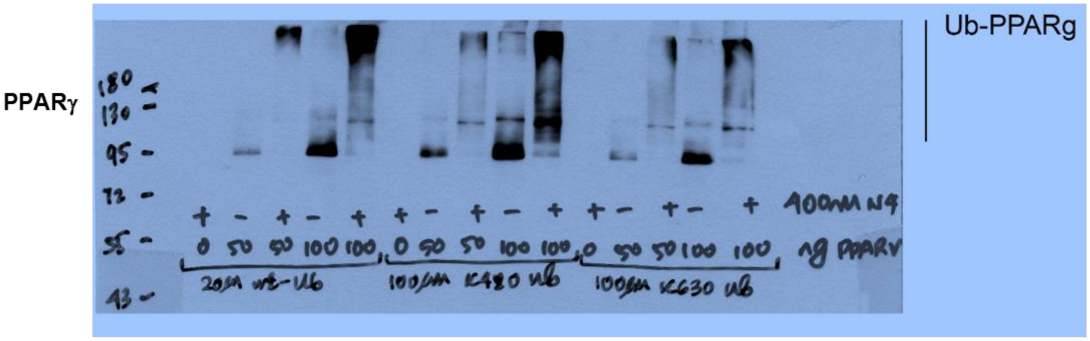

Fig 4A

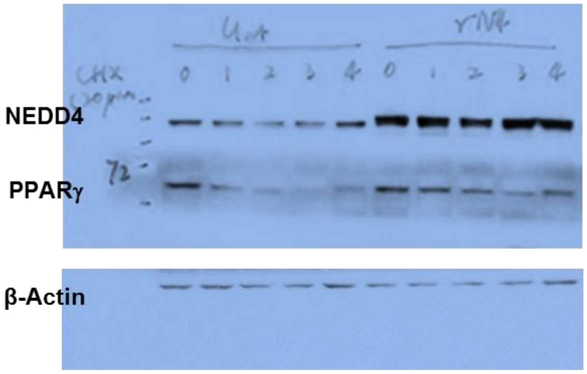

Fig 4B

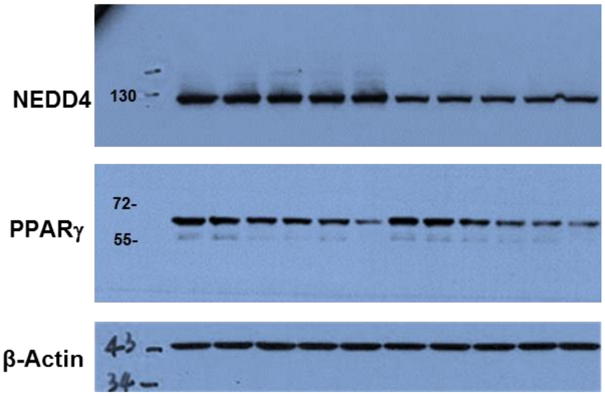

Supplementary Figure S4 continued

Fig 4C

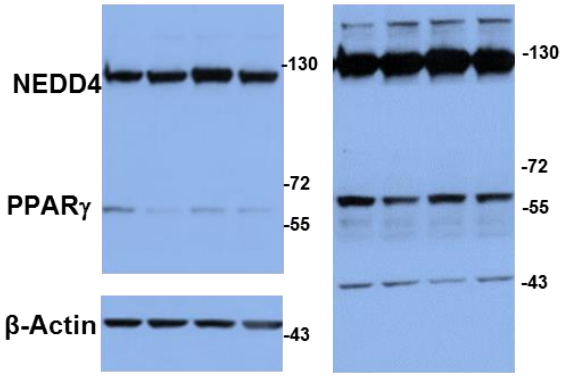

Fig 4E

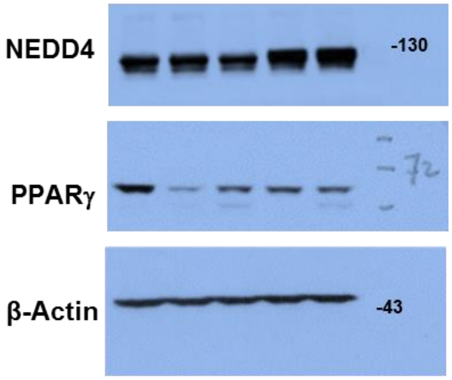

Fig 4D

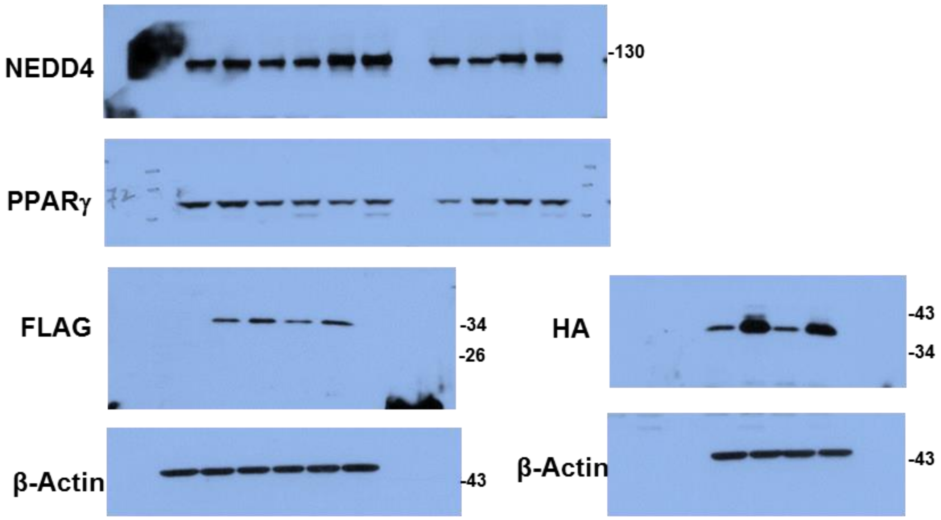

Fig 5B

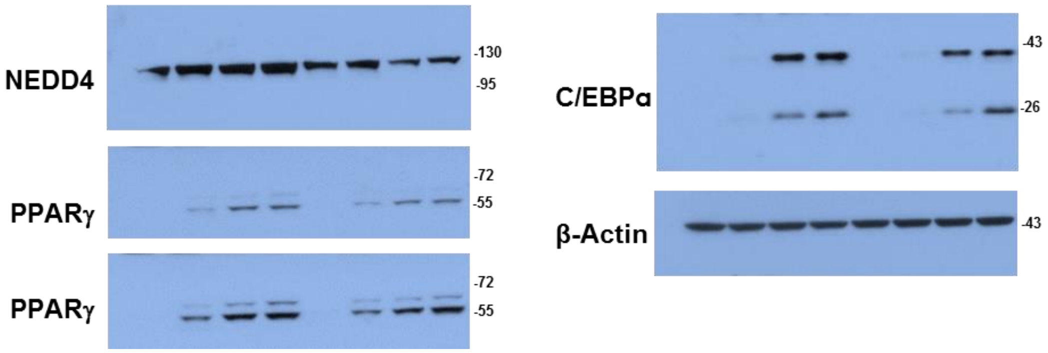

Supplementary Figure S4 continued

Fig 6A

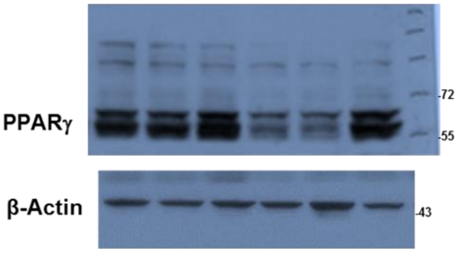

Fig 6B

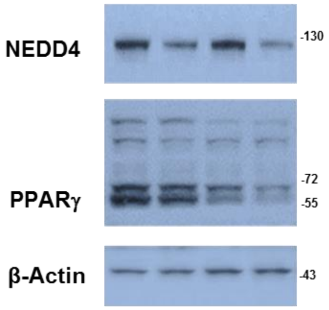

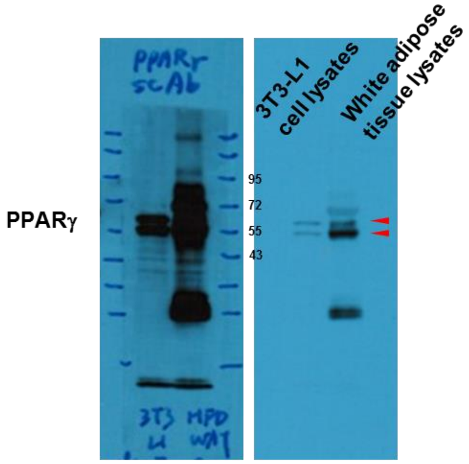

Fig 6C

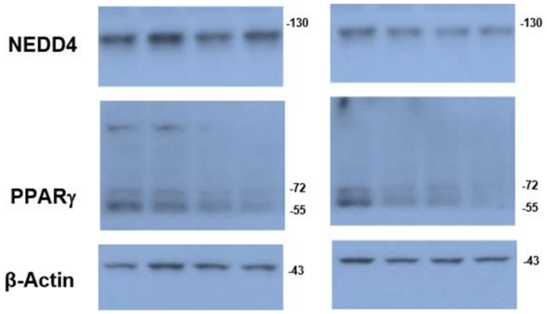

Fig 7C

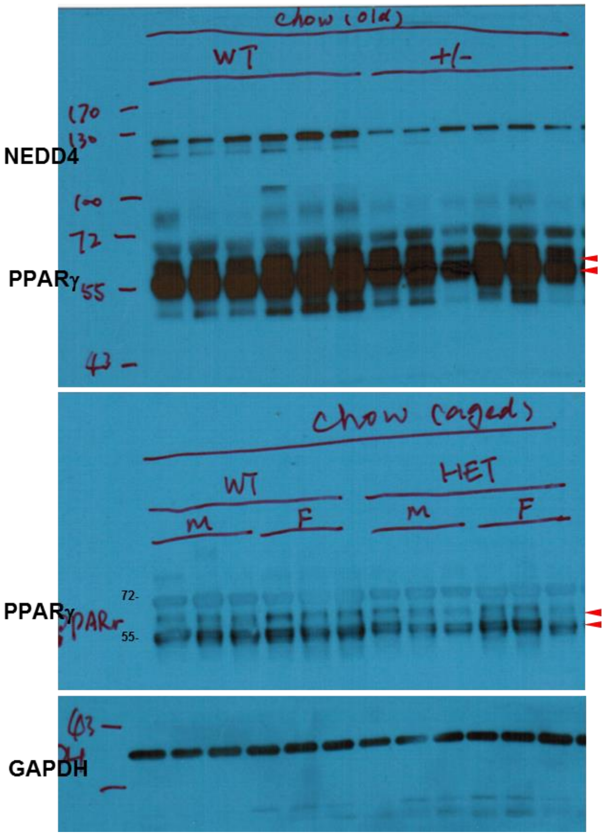

Fig 7D

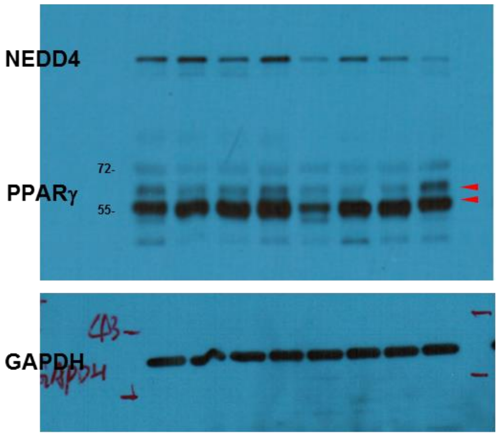

Supplementary Figure S4 continued
